# Clinical Usefulness of Novel Serum and Imaging Biomarkers in Risk Stratification of Patients with Stable Angina

**DOI:** 10.1155/2014/831364

**Published:** 2014-06-19

**Authors:** George Tsaknis, Iraklis Tsangaris, Ignatios Ikonomidis, Argirios Tsantes

**Affiliations:** ^1^Department of Respiratory Medicine, Glenfield Hospital, University Hospitals of Leicester, Groby Road, Leicester LE3 9QP, UK; ^2^Second Department of Critical Care Medicine, Attikon University Hospital, University of Athens, Medical School, 1 Rimini Street, Haidari, 12462 Athens, Greece; ^3^Second Department of Cardiology, Attikon University Hospital, University of Athens, Medical School, 1 Rimini Street, Haidari, 12462 Athens, Greece; ^4^Laboratory of Haematology and Blood Bank Unit, Attikon University Hospital, University of Athens, Medical School, 1 Rimini Street, Haidari, 12462 Athens, Greece

## Abstract

Inflammatory mediators appear to be the most intriguing yet confusing subject, regarding the management of patients with acute coronary syndromes (ACS). The current inflammatory concept of atherosclerotic coronary artery disease (CAD) led many investigators to concentrate on systemic markers of inflammation, as well as imaging techniques, which may be helpful in risk stratification and prognosis assessment for cardiovascular events. In this review, we try to depict many of the recently studied markers regarding stable angina (SA), their clinical usefulness, and possible future applications in the field.

## 1. Introduction

Angina is chest discomfort caused by myocardial ischemia without necrosis, further qualified by its precipitating factors, time course to relief, and clinical characteristics, such as pain radiation and quality. Typical angina may be triggered by increased activity (exercise, sexual activity), emotional stress (anger, fright, or stress), or cold, wind, and fever. The discomfort of exertional angina is relieved by rest within 1–5 min or more rapidly with sublingual nitroglycerin and attacks usually last from 2 to 10 min. Characteristically, there is heaviness or pressure retrosternally, with possible radiation to the ulnar aspect of the left arm, neck, jaw, midabdomen, right arm, or shoulders. The average frequency of angina attacks in patients is about 2 per week. Many patients voluntarily cut back their activities to avoid further episodes. Clinically, chronic stable angina (SA) is generally caused by one or more significant obstructive lesions in coronary arteries, defined as stenosis of >50% of the diameter of the left main coronary artery or stenosis of >70% of the diameter of a major epicardial vessel. Precipitating circumstances remain similar between episodes, thresholds may be predicted by patients, and relief patterns become known. Since stenoses are fixed, the angina is due to demand ischemia and seems to be the most common symptom in patients with coronary artery disease (CAD).

**Figure 1:**
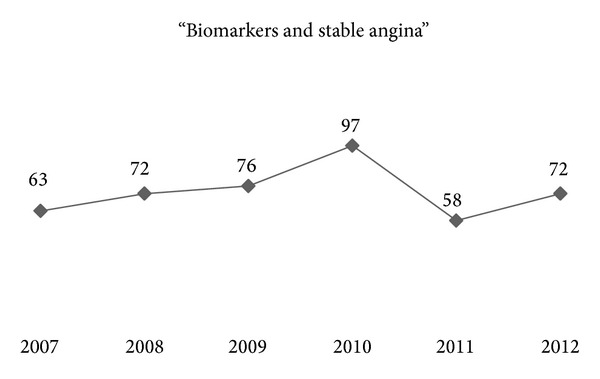
Distribution of PubMed search results within the last 6 years, per calendar year, with the search terms “biomarkers AND stable angina.”

**Figure 2:**
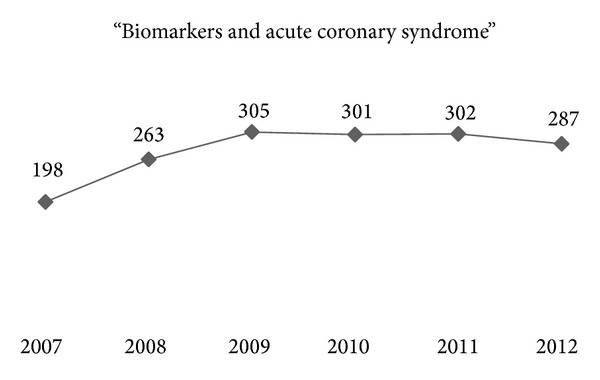
Distribution of PubMed search results within the last 6 years, per calendar year, with the search terms “biomarkers AND acute coronary syndrome.”

Almost 7 million Americans suffer and 400,000 new cases are added each year, resulting in very high economic burden estimated at 1.3% of the NHS budget in the UK and $75 billion in 2000 in the USA [1, 2]. Interestingly, real-life data on clinical outcome in SA outside randomized controlled trials are lacking, and in recent clinical trials the annual mortality ranges from 0.9% to 2.9%. There is growing interest in the last 6 years on risk stratification in SA patients specifically; hence risk factor research inevitably followed this concept of individualization (Figures 1 and 2).

Recently, the Euro heart survey for SA [3], after recruiting more than 3,000 patients, determined the clinical and investigative factors to predict death or AMI in patients suffering from SA and also developed a prediction model to assist in prognostication of patients with a clinical diagnosis of SA. The presence of any comorbidity, such as diabetes, the severity of angina, shorter duration of symptoms, left ventricular dysfunction, and ST changes on the resting ECG, independently predicted outcome. The predictive model involved these six characteristics to estimate the probability of death or AMI within the year after presentation with SA. This model was found to be simple and objective and allowed discrimination between an extremely low risk population (rate of death and nonfatal infarction per year, <0.5%) and patients at high risk over the one-year study period. Its predictive validity was comparable to older models and more importantly was relevant in real-life cases, in contrast with the highly selected populations reported in past randomized controlled studies. In this contemporary evaluation of the prognosis associated with SA, the incidence of death and myocardial infarction was 2.3/100 patient-years. These findings add to the existing published data by Rapsomaniki et al. [4] on the CALIBER prognostic models, which incorporated real-life clinical characteristics highlighted by the 2012 ACCF/AHA [5] and the 2006 ESC guidelines [6] for the initial evaluation, such as deprivation, atrial fibrillation, cancer, liver disease, depression, anxiety, and haemoglobin, factors that have not previously been incorporated in prognostic models for stable CAD, hence making the outcome data clinically relevant. In line with the above is the data from the Swedish study group in SA [7], reporting that easily accessible clinical and demographic variables provide a good risk prediction in SA. These variables were age (1.04 per year [1.00–1.08], *P* = 0.041), female sex (0.33 [0.16–0.69], *P* = 0.001), fasting blood glucose (1.29 per mM [1.14–1.46], *P* < 0.001), serum creatinine (1.02 per *μ*M [1.00–1.03], *P* < 0.001), and leucocyte counts (1.21 per 106 cells/L [1.06–1.40], *P* = 0.008). Impaired glucose tolerance and an elevated serum creatinine were found to be particularly important.

In this review article, we try to broach into the majority of the novel biochemical (Table 1) and imaging risk factors related to SA, balancing disease-oriented evidence (DOE) as well as patient-oriented evidence that matters (POEM).

### 1.1. Pathophysiology

**Figure 3:**
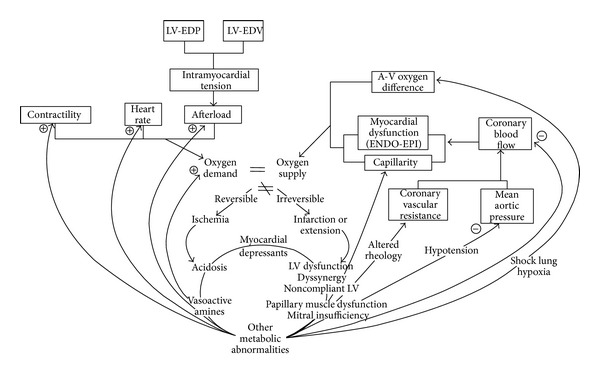
Schematic approach of the current established mechanisms in stable angina pathophysiology (LV: left ventricle; LV-EDP: left ventricular end-diastolic pressure; LV-EDV: left ventricular end-diastolic volume).

The inability of the coronary arteries to increase blood flow in response to increased cardiac metabolic demands is the baseline dysfunction in SA. Normally, coronary endothelium excretes nitric oxide (NO) from its cells as a response to physical activity or any other demanding cardiac effort. Atherosclerosis damages the endothelium and makes endothelial cells permeable to cholesterol as well as other harmful substances, resulting in dysfunctional NO release and atherosclerotic plaque formation. In patients with stable CAD, the process of atherosclerosis involves a fundamentally different histopathology compared with ACS or UA. In chronic stable CAD, we have the formation of a small lipid core with a very thick fibrous cap and a low proclivity to rupture, causing narrowing of the arterial lumen as time goes by and producing symptoms, whereas in ACS/UA the principal histopathologic picture is that of a large lipid core subtended by a thinned, inflamed cap, which harbors the high-risk or vulnerable plaque with a high proclivity for rupture. When these plaques rupture or suffer “fissuring,” clot formation takes over (less in stable CAD, more in ACS/UA) with the usual acute ischemic consequences. The type of exposed substrates to circulation plays a major role in thrombosis formation, as platelets adhere more to exposed collagen and not to foam cells (as in SA). It has been recognized that myocardial ischemia results from an imbalance between myocardial energy supply, from insufficient sources of oxygen and substrate (glucose, free fatty acids), and myocardial oxygen demand. Usually this is simply referred to as an imbalance between myocardial oxygen supply and demand, but it should be clear that substrate supply, utilization, and enzymatic activities, along with other variables involved in metabolism and mitochondrial function, play a major role in the pathogenesis of myocardial ischemia in SA and ACS and during reperfusion ischemic injury. Many of the global relationships and positive feedback loops relating to the inequality of myocardial oxygen supply and demand have not changed in many years, although molecular, electrophysiological, conceptual, and technological advances have been changed considerably. Myocardial energy imbalance is central to all ischemic syndromes: SA, AMI, and cardiogenic shock. The variables determining myocardial oxygen supply are altered by negative feedback loops from complications of poor left ventricular function. Those factors affecting myocardial oxygen demand (heart rate, afterload, preload, and contractility) are altered by positive feedback loops from those events perpetuating systemic features. An increase in left ventricular end-diastolic pressure (LV-EDP) or volume (LV-EDV) increases preload according to Laplace's Law. Both negative feedback on oxygen supply and positive feedback on oxygen demand tend to increase the inequality between the two and may jeopardize poorly perfused myocardial tissue (Figure 3). When ischemia progresses beyond the reversible stage of angina and myocardial necrosis follows, well-known hemodynamic, metabolic, and mechanical sequelae may occur.

### 1.2. Current Use of Circulating Biomarkers in CAD

During the past decades, various types of serum marker levels were widely used in the risk management of CAD. Mainly, these were markers of myocardial necrosis, such as aspartate transaminase in the 1950s, creatinine kinase (CK) in the 1960s, CK-MB in the 1970s, and troponins in the 1980s, primarily used as diagnostic tests with high negative and positive predictive value. Cardiac troponins are a clear example in clinical medicine where urgent clinical decision and marker measurement are closely related. Although a vast variety of other markers are routinely checked among patients with CAD, their true clinical use in terms of decision-making is not clear. As an example, serum creatinine has been estimated among people with suspected CAD for decades, but only in the last decade has its potential prognostic value been considered.

In patients with SA, circulating biomarkers have been recommended as potentially useful in risk stratification. As an example, the Centers for Disease Control/American Heart Association statement for health-care professionals recommended that one biomarker among SA patients (C-reactive protein, CRP) may be useful as an independent prognostic marker. On the other hand, there is a variability observed between clinicians and centers in which biomarkers are evaluated among SA patients, and only anecdotal evidence exists for biomarker use in common everyday clinical practice other than clinical studies.

There are various possible pathophysiologic mechanisms by which these markers may interfere with prognosis in SA patients, but this is of secondary importance taking into account the urgent clinical decision-making. The primary issue is to understand, if possible, each responsible mechanism of risk prediction and secondarily which marker is better.

## 2. Biomarkers and Stable Angina

### 2.1. Pro- and Anti-Inflammatory Markers

#### 2.1.1. High Sensitivity C-Reactive Protein (hs-CRP)

In older men and women, elevated CRP has been associated with an increased 10-year risk of CAD, regardless of the presence or absence of other common cardiac risk factors [8, 9]. A single CRP measurement has been shown to provide information beyond conventional risk assessment, especially in intermediate-Framingham-risk men and high-Framingham-risk women. Elevated hs-CRP has been previously related to the amount of necrotic core in the culprit lesion in SA patients. In a study by Kubo et al. [10], the percentage of necrotic core was significantly greater in the elevated hs-CRP group compared with the normal hs-CRP group (20 ± 9 versus 16 ± 8%, *P* = 0.014). The percentage of necrotic core was positively correlated with the serum hs-CRP level (*r* = 0.20, *P* = 0.037). Further studies are needed to determine risk prediction ability of this marker, with clearer description of the study population and variable adjustment for simple clinical risk factors, such as age, sex, smoking habits, diabetes, obesity, and lipid panel abnormalities.

#### 2.1.2. Growth and Differentiation Factor- (GDF-) 15

It is a cytokine involved in cell-differentiation and embryogenesis and belongs to the superfamily of proteins called “*transforming growth factor-beta family*” along with activins and inhibins [11]. Normally, GDF-15 shows high expression in placental tissue and a very low expression in normal tissue. However, GDF-15 levels are notably increased in various stress conditions, including ACS [12–14]. In addition, there is a sense that GDF-15 levels might reflect unique additional information about cardiac risk in general other than just increased inflammatory-induced protein activity. This is supported by data showing that GDF-15 correlates positively with body mass index (BMI) and also relates independently with CRP and NT-proBNP regarding ACS populations [12, 15].

A large-scale study regarding the use of GDF-15 levels in SA patients published by Schaub et al. [16] showed that when circulating serum GDF-15 levels measurement was added to a clinical risk predictive model regarding CAD mortality, the predictive accuracy improved significantly (from AUC = 0.74 to AUC = 0.85, *P* = 0.005). In a subgroup of 757 SA patients, GDF-15 levels remained independently associated with mortality, even when adjusted for left ventricular ejection fraction (LVEF) (*P* < 0.001). In a recently published, prospective, international multicenter study, GDF-15, high-sensitivity cardiac troponin T (hs-cTnT), and B-type natriuretic peptide (BNP) were measured in 646 random patients presenting with acute chest pain to the emergency department. In this study, GDF-15 predicted all-cause mortality independently of and more accurately than hs-cTnT (AUC 0.85 (95% CI 0.81–0.90) versus 0.77 (95% CI 0.72–0.83), *P* = 0.002) and BNP (AUC 0.75, 95% CI 0.68–0.82, *P* = 0.007) but did not seem to help in earlier AMI diagnosis [17].

Our suggestion is that these findings, albeit novel and useful, have to be validated by more studies and also different researchers, in a multicenter basis, because most of the available data has been reported by the same research group.

#### 2.1.3. Neopterin

Neopterin is a marker of macrophage activation, atherosclerotic plaque progression, fibrous cap disruption, and intracoronary thrombus formation. It is a pteridine derivative and a byproduct of the guanosine triphosphate-biopterin pathway. Neopterin has been studied in the concept of discovering a connection between the inflammatory process and left ventricular (LV) function, as depicted by left ventricular ejection fraction (LVEF) [18]. In recent published data regarding SA patients, increased neopterin levels showed inverse correlation with LVEF values and high neopterin levels were found to be an independent predictive factor for LV dysfunction (LVEF < 45%) (OR 8.52, CI 95% 1.10–65.64; *P* = 0.040). Receiver operating characteristic analysis for neopterin showed an AUC of 0.736 (CI 95% 0.59–0.87, *P* < 0.009) for prediction of LV dysfunction [19] concluding that neopterin could be of clinical value for risk stratification in these patients.

#### 2.1.4. Interleukin-6 (IL-6)

Interleukin-6 is a 22–27 kD glycoprotein secreted by activated monocytes, vascular smooth muscle cells, and adipose tissue and acts as both an inflammatory and anti-inflammatory cytokine in response to a stressful insult of any kind such as trauma, infection, and burns. Inflammation has been accepted to play a role at all stages of atherosclerotic CAD including progression and rupture of the plaque [20, 21]. Additionally, the discovery that cytokine production is elevated not only in ACS but also in patients suffering from SA may indicate prolonged duration of inflammatory processes in vascular wall [22].

This cytokine is studied in relationship between other biomarkers and conventional risk factors in order to assess its clinical value. In a recent study including 34 patients with SA, levels of IL-6 were correlated with severe stenosis of the left anterior descending artery (LAD) and a higher Gensini score (as an objective score of CAD severity). Interestingly, when patient groups were compared, STEMI and NSTEMI groups had significantly higher IL-6 levels than the SA group (*P* = 0.002; *P* = 0.005, resp.). The sensitivity and specificity for IL-6 as a CAD prediction marker were 46% and 86%, respectively, which led the investigators to conclude that the use of IL-6 levels alone could be useful in ruling out CAD [23]. In other studies, higher IL-6 levels were found in patients who had already experienced UA when compared with patients with SA [24–26]. In the PRIME study [27], IL-6 levels showed their value for predicting SA or ACS over a 5-year followup. To our knowledge, this was the first population-based observational study comparing systemic inflammatory mediators in predicting SA in a previously healthy population.

Larger studies combining objective coronary angiographic parameters and histologic findings may be helpful in evaluating the use of IL-6 as risk predictor.

#### 2.1.5. Interleukin-10 (IL-10)

Interleukin-10 is not a new member in ACS research, but there is growing controversial literature regarding its prognostic value. This cytokine is mainly expressed in monocytes and type 2 T helper cells (T_H_2), mast cells, CD4+CD25+Foxp3+ regulatory T cells, and a certain subset of activated T cells and B cells. Recent published data in Nature Medicine showed that IL-10 can also be produced by monocytes upon programmed-death ligand (PD-L1, PD-L2) triggering in these cells [28]. The existing experimental and human data suggests that the PD-1/PD-L1 and PD-L2 pathways play a key role in controlling the immune response of the proatherogenic T cell immunity, associated with the pro- and anti-inflammatory process [29–32]. More specifically, the expression of PD-1 and PD-L1 is significantly downregulated on T cells and myeloid dendritic cells (mDCs) in CAD patients compared to healthy individuals [31]. In a prospective study with 5-year followup, elevated baseline IL-10 levels were found to be an independent predictor of long-term adverse cardiovascular outcomes in ACS patients [33].

#### 2.1.6. Myeloperoxidase (MPO)

It is a 150 kD peroxidase enzyme stored in azurophilic granules of the neutrophil, secreted at sites of inflammation, interfering in the pathway of cell oxidation, and has a well-documented role in atherosclerotic disease, in terms of plaque progression and vulnerability, along with matrix metalloproteinases (MMPs) [34–37]. In culprit coronary lesions of SA patients, MPO-producing cells were found to be lower in concentration and less frequent, compared with ACS patients [38–43].

In a 3,000 patients' population study, high levels of MPO were an independent predictive risk factor for developing CAD in healthy individuals (OR for the highest quartile of MPO 1.36, 95% CI 1.07–1.73) [37]. In addition, in a different study, MPO did not show significant difference between the control (24.2 ± 5.7 *μ*g/L) and SA groups (26.3 ± 4.8 *μ*g/L), but plasma MPO levels in patients with ACS (93.6 ± 20.3 *μ*g/L) were significantly higher than in patients with stable angina and the healthy control subjects (*P* < 0.05) [44]. Furthermore, in a recent study, there was no significant difference in serum MPO concentrations between patients with SA and controls. Additionally, in the same study, serum MPO levels were significantly higher in AMI and UA patients compared with SA (both *P* < 0.001), but there was no difference between AMI and UA. At followup, the mean MPO concentrations had significantly decreased in patients with SA (*P* = 0.008), UA (*P* < 0.001), or AMI (*P* < 0.001) and controls (*P* < 0.001). These findings are in contrast to data showing increased concentration of plasma MPO levels in patients with SA or ACS or in some cases no difference between SA and controls [39, 45–47].

Direct comparison of MPO levels between studies is obtrusive, because the sampling and laboratory assays for MPO levels seem to differ. In conclusion, this data suggests that MPO is a powerful marker of acute coronary inflammation and also a strong mediator for neutrophil activation. As research groups remain in controversy, we need more data to integrate the use of MPO in everyday clinical practice.

#### 2.1.7. Interleukin-17 (IL-17)

Interleukin-17 is a 155-amino acid protein that is a disulfide-linked, homodimeric, secreted glycoprotein with a molecular mass of 35 kD. It is a potent mediator in delayed-type reactions by increasing chemokine production in various tissues to recruit monocytes and neutrophils to the site of inflammation. Interestingly, IL-17 bears no resemblance to any other known proteins or structural domains [48, 49].

The role of IL-17 in SA or CAD remains under investigation. It is established that Th17 cells producing IL-17 are involved in the pathogenesis of atherosclerosis inducing vascular endothelial cell apoptosis, but the exact pathway is not clear [50–54]. The hypothesis, which is supported by limited data, is that IL-17 is secreted late on the inflammatory cascade, along with MPO, and attracts adhesion molecules (i.e., intercellular adhesion molecule (ICAM)) which are involved in ACS and have a role in coronary inflammation [50, 55].

In a small population study [44], IL-17 levels were compared among patients with ACS and no statistical difference was found between the SA and the control group (2.3 ± 0.38 pg/mL versus 2.2 ± 0.22 pg/mL, resp.). The important finding in this study was the correlation between plasma MPO and IL-17 levels in all study participants (*R* ^2^ = 0.9110, *P* < 0.05), supporting the hypothesis that IL-17, as MPO, is a powerful indicator of acute coronary inflammation.

#### 2.1.8. Stromal Cell-Derived Factor-1 (SDF-1; CXCL-12)

The stromal cell-derived factor-1 (SDF-1) is a small cytokine that belongs to the larger family of intecrines, chemokines that can be classified into two subgroups, the CC and the CXC family, with SDF-1 belonging to the latter. It is secreted in response to any vascular injury or ischemia and regulates recruitment of CXCR4+ cells on the vascular wall and there is evidence for its crucial role in tissue regeneration and revascularization, reflecting a possible cardioprotective effect after myocardial infarction* in vivo* [56–58].

When SDF-1 was compared with classic cardiovascular risk factors such as arterial hypertension, diabetes, smoking, or hyperlipidemia, there was no association found and no correlation with any biochemical parameter (except an inverse correlation with cholesterol levels, *P* = 0.035), either in the whole study population or in the SA group, was found [59]. Additionally, there was no statistical difference in SDF-1 levels between the NSTEMI and the SA group. In a recent study regarding the expression of SDF-1 in nonvalvular paroxysmal or permanent atrial fibrillation, patients with SA had an impaired expression of SDF-1 compared with patients with ACS [59], which is in line with previously reported findings by Stellos et al. [60], showing increased platelet-bound-SDF-1 in patients with SA and paroxysmal atrial fibrillation (AF), compared to patients on sinus rhythm or persistent/permanent AF (*P* < 0.05 for both), and patients with ACS presented with enhanced platelet-bound-SDF-1 compared with SA.

Based on currently available data, SDF-1 can discriminate SA from ACS in the presence of nonvalvular arrhythmias, but not SA from acute ischaemic episodes* per se*, when serum levels are being measured.

#### 2.1.9. Procalcitonin (PCT)

Procalcitonin is a peptide precursor of calcitonin, composed of 116 amino acids and produced by parafollicular cells (C cells) of the thyroid gland and by the neuroendocrine cells of the lung, intestine, and liver. It is a well-established biomarker in critically ill patients, in terms of predicting mortality, sepsis, and septic shock development, distinguishing bacterial from nonbacterial infections and being helpful in reducing unnecessary antibiotic therapy [61, 62]. In CAD, inflammatory response and ischemic damage can lead to PCT production, which is supported by data implicating PCT as a novel biomarker for AMI [63]. Moreover, PCT has previously demonstrated good correlation with the extent of atherosclerosis and has been associated with an adverse outcome [64–66]. For SA, its utility is investigated only in recent years.

Recently [67], PCT was evaluated in a total of 1,300 subjects with SA, among a large cohort of CAD patients. Patients with ACS had increased PCT levels compared to the SA group (0.016 (0.011/0.027) ng/mL versus 0.014 (0.009/0.014) ng/mL; trend *P* < 0.0001). There was an association of significantly increased PCT levels and classical risk factors, such as male sex (*P* < 0.0001), diabetes (*P* < 0.0001), and BMI > 30 (*P* < 0.0001). In terms of mortality, increased PCT levels at baseline were related to higher cardiovascular mortality (*P* = 0.00018) and higher cardiovascular event rate (*P* = 0.026) and also independently related to future cardiovascular death (HR: 1.34; 95% CI: 1.08–1.65; *P* = 0.0070) when adjusted for clinical variables. On the other hand, when PCT was adjusted for CRP, its association with mortality was lost.

Serum PCT levels might be a representative marker for the patients' inflammatory status and could be used for risk stratification in CAD, but there are few available data regarding SA.

#### 2.1.10. Fetuin-A

Fetuin-A has been recognized as an anti-inflammatory cytokine and modulator in the atherosclerotic process [68]. Its role in cardiovascular disease has been previously investigated, in a cohort from the European Prospective Investigation into Cancer and Nutrition (EPIC)-Potsdam Study [69], and linked to an increased risk of AMI (as well as stroke) in patients with elevated fetuin-A serum levels. In a study by Bilgir et al. [70], fetuin-A levels have been found decreased in SA patients presenting with chest pain, compared to controls, but higher than in patients with AMI. As far as AMI outcomes are concerned, an increased fetuin-A in serum has been associated with an excellent survival rate (NPV = 97% overall) [71] even in high-risk populations, suggesting a sound pathogenetic role in the ischaemic event.

#### 2.1.11. Lipoprotein-Associated Phospholipase A_2_ (Lp-PLA_2_)

This 50 kDa protein is a phospholipase A_2_ enzyme that is encoded by the PLA_2_G_7_ gene. It belongs to the family of platelet-activating factor acetylhydrolases, known to participate in atherogenic process, notably in complex plaques [72–74].

There is growing data regarding the positive correlation of Lp-PLA_2_ levels and cardiovascular risk. In the West of Scotland Coronary Prevention Study (WOSCOPS), almost 6,600 hyperlipidemic middle-aged males were followed up for 5 years and inflammatory markers were measured. The strongest predictor of an adverse cardiovascular outcome was Lp-PLA_2_, independently from traditional markers such as CRP (relative risk of 1 SD increase = 1.18, 95% CI: 1.05–1.33, *P* = 0.005) [75–77]. Regarding ACS, in the PEACE trial, Serruys et al. showed that in patients with stable CAD elevated Lp-PLA_2_ and hs-CRP levels were significant predictors of acute coronary syndromes (*P* < 0.005 and 0.001, resp.). In addition, Lp-PLA_2_ was the only significant predictor for coronary revascularization during followup [78]. In a very recent study by Ikonomidis et al. [79] that evaluated 111 angiographically confirmed stable CAD patients, Lp-PLA_2_ was positively associated with carotid intima-media thickness (CIMT), and in the multivariate analysis Lp-PLA2 was an independent determinant of reactive hyperemia using fingertip peripheral arterial tonometry (RHI-PAT), coronary flow reserve (CFR), CIMT, and pulse wave velocity (PWV) in a model including age, sex, smoking, diabetes, dyslipidemia, and hypertension (*P* < 0.05 for all vascular markers). During a 3-year followup, Lp-PLA2, RHI-PAT, and CFR were independent predictors of cardiac events in this CAD cohort. Overall, elevated Lp-PLA2 concentration was related to endothelial dysfunction, carotid atherosclerosis, impaired CFR, increased arterial stiffness, and adverse outcomes in stable CAD. These findings suggest that the prognostic role of Lp-PLA2 in chronic CAD can be proved helpful in clinical practice. Moreover, Lp-PLA_2_ has been recently promoted as a novel therapeutic target [79, 80]. When darapladib, the specific inhibitor of Lp-PLA_2_, was added to statin therapy in patients with known CHD, there was a reduction in inflammatory markers such as CRP and IL-6, indicating a synergistic effect in inflammation amelioration. In a study by Galis and Khatri [81], darapladib was evaluated for its effect on the vascular wall, in patients with proven CAD by angiography. In a dose of 160 mg daily, darapladib decreased the necrotic core expansion significantly (−0.5 ± 13.9 mm^3^; *P* = 0.71 in the darapladib arm). Currently, two large-scale ongoing trials will try to show a beneficial effect of Lp-PLA_2_ inhibition (STABILITY and SOLID-TIMI 52) and therefore depict a new therapeutic target in patients with CAD. Mortality outcomes from these cohorts will show the need for a new drug or the need for more laboratory and clinical research on the field.

#### 2.1.12. Matrix Metalloproteinases

Matrix metalloproteinases (MMPs) are zinc-dependent endopeptidases that belong to a larger family of proteases known as the metzincin superfamily. They are incriminated for plaque development in atherosclerotic disease and also in plaque rupture and subsequent atherothrombosis [82–89].

The levels of MMPs have been consequently evaluated in different CAD patients, including SA and ACS. In a recent study, levels of both MMP-2 and MMP-9 were significantly higher in patients with ACS compared to SA or healthy controls with normal coronary arteriography, which might indicate that the release of these two MMPs is related to the pathophysiology of ACS only [90]. Additionally, in another study [91], levels of MMP-8 and MMP-9 in plasma did not correlate with any common risk factor, such as waist circumference or smoking, but were highly correlated to MPO (both *R* ^2^ = 0.80, *P* < 0.001). In the same study, neutrophils of SA patients released more MMP-9 in response to IL-8 than controls. In agreement with a number of previous studies [92, 93], there were no significant differences in circulating levels of MMP-9 between SA patients and controls. Interestingly, plasma levels of MMP-8 differ between SA patients and controls which is in contrast with previous studies [94, 95] that have shown raised plasma MMP-8 in SA patients.

In conclusion, since the neutrophil release of MMP-9 is thought to be an early marker of neutrophil activation, these findings may depict a persistent neutrophil activation in SA patients but not clarify MMPs value in risk stratification.

#### 2.1.13. Tissue Inhibitors of Metalloproteinase (TIMP)

They are the main regulators of matrix metalloproteinase activity and compromise a family of four protease inhibitors, TIMP-1, TIMP-2, TIMP-3, and TIMP-4. The balance between TIMPs and MMPs is thought to be decisive for plaque stability. Interestingly, reduced amounts of TIMP-1 and TIMP-2 (the main endogenous regulators of MMP-8 and MMP-9 activity) have been reported in unstable atherosclerotic lesions compared to stable atherosclerotic lesions [96].

There is very limited and also controversial data regarding SA patients, with a few clinical studies reporting increased plasma levels of TIMP-1 in SA patients [97], while others show levels similar to healthy subjects [92]. Likewise, the clinical impact of circulating TIMP-2 levels has been conflicting. Therefore, so far we can only theorize about the effects of high levels of TIMPs in SA. Their potential implications remain to be clarified in future studies.

### 2.2. Cardiovascular Function and Remodeling

#### 2.2.1. C-Terminal Provasopressin (Copeptin)

Copeptin is the C-terminal of provasopressin, composed of 39 amino acids and secreted from neurohypophysis in response to stimuli (hemodynamic or osmotic type). It has been recently proposed by several study groups as an early marker of AMI risk stratification and prognosis in chronic heart failure [98–106]. There are few available data about copeptin and its prognostic value in SA patients.

In a large cath lab cohort (2,700 patients; SA group *n* = 1, 384) [107], copeptin was evaluated for its prognostic value regarding morbidity and mortality. Interestingly, patients with a family history of CAD had significantly higher copeptin baseline levels (*P* = 0.0141). A Kaplan-Meier analysis showed that patients with increased copeptin levels (serum level ≥ 20.4 pmol/L) suffered more events of the combined primary endpoint and of all-cause death alone at 90 days, compared to patients with lower levels. However, despite the promising data, we note that the primary endpoint of this study was a combined adverse outcome endpoint, which is of limited value compared with a mortality outcome alone.

In short, copeptin may be a useful prognostic tool for the prediction of major adverse cardiovascular events such as AMI, stroke, and all-cause mortality in CAD patients, but these findings cannot be extrapolated in SA. Further studies should investigate copeptin exclusively in SA patients and the optimal cutoff value.

### 2.3. MicroRNAs

MicroRNAs (also known as miRs or miRNAs) are RNAs of a non-coding molecule approximately 25-NT-long, that negatively regulate gene expression by binding to 39 untranslated regions of targeted messenger RNAs [108]. They have been found to be involved in many biological processes, from cellular differentiation, proliferation [109, 110], cell death, apoptosis [111, 112], and synaptic plasticity [113] to immunity [114] and cardiovascular development [115], as well as cardiovascular diseases [116, 117].

In a study by Latronico and Condorelli [118] that examined circulating miRNA expression in plasma of patients with CAD compared to controls, aiming to identify novel biomarkers in SA and UA, ROC curve analyses showed a good diagnostic potential (AUC ≥ 0.85) for miR-1, miR-126, and miR-483-5p in patients with SA. Moreover, cluster analysis showed that the combination of miR-1, miR-126, and miR-485-3p in SA correctly classified patients compared with controls, with an efficiency of ≥87%. Interestingly, none of the investigated combinations of miRNAs was able to reliably discriminate SA from UA patients. Moreover, the study showed that specific plasmatic miRNA signatures have the potential to accurately discriminate patients with angiographically documented CAD from matched controls.

Further studies are needed, with larger populations, to address the potential utility of plasmatic miRNAs as biomarkers of SA, as well as to clarify the mechanisms of their release in serum.

### 2.4. Imaging

Compared to a simple exercise electrocardiography testing (XECG), perfusion imaging with ^201^Thallium or ^99m^Technetium-sestamibi raises sensitivity, but prognostic value is less established [119]. Perfusion imaging is particularly useful when the resting ECG is abnormal, specifically in women because of false positive results on XECG [120]. In symptomatic patients who have had prior revascularization, reversible areas of ischemia may be quantified and localized to specific areas of the myocardium [121]. ^99m^Technetium-sestamibi produces better and faster images with decreased attenuation, has lower sensitivity for viable myocardium than ^201^Thallium, and is more expensive. Increased lung uptake after testing, left ventricular dilation, and multiple perfusion defects are associated with left main coronary or severe multivessel disease and should be followed by coronary angiography. Patients with two or more perfusion defects and ventricular dysfunction are also candidates for angiography. Perfusion imaging as a single test has been found to lower rates of hospital admission by up to 52% while evaluating acute chest pain in the emergency department [122]. A number of differences in plaque density between patients with SA and AMI have been reported using optical coherence tomography (OCT) imaging to assess plaque vulnerability [123]. Survivors of AMI who were undergoing percutaneous interventions and those with stable lesions in multiple vessels had OCT images performed of infarct-related lesions or lesions slated for revascularization, as well as non-infarct-related and nontarget lesions. Images from OCT study found intracoronary thrombus in all patients suffering an AMI, and none in patients with SA. A ruptured coronary plaque was identified in 77% of AMI patients, but only in 7% of SA patients, suggesting differences in plaque pathophysiology.

With the increasing use of hybrid single photon emission computed tomography (SPECT/CT) devices, myocardial perfusion imaging (MPI) and coronary artery calcium (CAC) scoring can be easily combined and performed in a single session. However, in symptomatic patients with a very high CAC score, it is still unclear if MPI will provide any benefit in terms of the resulting implications for treatment as well as short-term prognosis. In a recent study by Prescott et al. [124] in patients with a low/intermediate risk of a coronary event with suspected but unconfirmed CAD and a high CAC score (≥1,000), ischaemia on MPI was a strong predictor for coronary revascularization. However, nonischaemic MPI does not exclude revascularization, and patients with persisting complaints should be considered for invasive angiography (OR 13.1; 95% CI: 7.1–24.3; *P* < 0.001). In the same study, patients who underwent scanning with the cadmium-zinc-telluride (CZT) gamma camera had fewer equivocal findings in SPECT (6% versus 18%, *P* = 0.002) and more often underwent stress only imaging (30% versus 16%, *P* = 0.0018).

In the ongoing iPOWER study [125], which was conducted to determine whether routine assessment of coronary microvascular dysfunction (CMD) in women with angina and no obstructive coronary artery disease is feasible and can identify women at risk, Doppler study and measurement of CFR of the left anterior descending artery was found to be feasible. At the end of this study that will recruit approximately 2,000 patients, more clear conclusions regarding the prognostic value of routine noninvasive techniques for microvascular function are expected.

In a recently published meta-analysis on the diagnostic accuracy and posttest outcomes of XECG and SPECT [126], compared with coronary computed tomography angiography (CCTA) in patients with stable angina, the per-patient sensitivity (95% CI) to identify significant CAD was 98% (93–99%) for CCTA versus 67% (54–78%) (*P* < 0.001) for XECG and 99% (96–100%) versus 73% (59–83%) (*P* = 0.001) for SPECT. The specificity (95% CI) of CCTA was 82% (63–93%) versus 46% (30–64%) (*P* < 0.001) for XECG and 71% (60–80%) versus 48% (31–64%) (*P* = 0.14) for SPECT. The OR of downstream test utilization for CCTA versus XECG/SPECT was 1.38 (1.33–1.43, *P* < 0.001), for revascularization 2.63 (2.50–2.77, *P* < 0.001), for nonfatal AMI 0.53 (0.39–0.72, *P* < 0.001), and for all-cause mortality 1.01 (0.87–1.18, *P* = 0.87). In a previously published study that compared CCTA with SPECT in patients with SA [127], patients who underwent a CCTA had increased incident of aspirin (22% versus 8%; *P* = 0.04) and statins use (7% versus −3.5%; *P* = 0.03) and similar rates of hospitalization related to CAD events and underwent more frequently an invasive coronary angiography or noninvasive cardiac imaging tests, and the majority underwent revascularization (8% versus 1%; *P* = 0.03). Significantly lower total costs were observed in the CCTA arm ($781.08 (interquartile range (IQR), $367.80–$4349.48) versus $1214.58 (IQR, $978.02–$1569.40); *P* = 0.001). Lower total estimated effective radiation dose was observed with CCTA (7.4 mSv (IQR, 5.0–14.0 mSv) versus 13.3 mSv (IQR, 13.1–38.0 mSv); *P* = 0.0001). Overall, CCTA proved to be better in guiding medical or revascularization therapy, with lower total cost and lower radiation exposure. Larger multicenter studies with longer followup, or meta-analyses of existing studies, are needed to fully comprehend the prognostic value of these modalities. In conclusion, both functional and anatomic assessment of CAD has prognostic value in SA. CCTA findings are strong predictors of future adverse events, with incremental value over clinical predictors, stress testing, and coronary calcification.

## 3. Conclusions

There is growing evidence suggesting that the use of a fixed marker panel combined with classical, easy, accessible data prior to testing may augment prognostic strength and accuracy in clinical practice [4, 7, 128, 129]. Based on current data, we believe that using a biomarker combination for risk stratification or mortality prediction, and adding an imaging study with incremental value over clinical predictors, stress testing, and coronary calcification such as CCTA, rather than a stand-alone marker, is the right clinical direction in SA.

Moreover, taking into account the very low reported mortality rates in SA, in the era of new available pharmacological agents (i.e., ranolazine) [130], a systematic evaluation of *s* concrete combination of biomarkers and imaging studies in a long-term, large-scale basis is deemed important in order to select patients that would benefit. Future research on microRNAs seems promising in clarifying the vague area of the inflammatory cascade in SA, bridging the pathophysiologic and clinical findings in order to predict outcomes effectively.

With the emergence of novel, sensitive biomarkers of inflammation, myocyte necrosis, vascular damage, and hemodynamic stress, it is becoming possible to characterize noninvasively the participation of different contributors in any individual patient. Although there are several novel biomarkers proposed for risk stratification in SA and our understanding for the specific biochemical role of each marker in the disease is still limited, it is plausible that elevated levels of circulating markers of inflammation reflect an intensification of focal inflammatory processes that destabilize vulnerable plaques.

Cardiac serum and imaging biomarkers provide a convenient and noninvasive means in clinical practice, in order to gain insights into the underlying causes and consequences of stable CAD that mediate the risk of recurrent or new events and may be targets for specific therapeutic interventions.

## Figures and Tables

**Table 1:** Summary of the most important data in this review, regarding biomarker use for risk stratification of SA patients.

Biomarker	Study	Comments
hs-CRP	Cushman et al., 2005 [8]	Elevated CRP levels were independently associated with increased 10-year risk of CHD in intermediate-Framingham-risk men and high-Framingham-risk women.

GDF-15	Kempf et al., 2009 [13]	GDF-15 remained an independent predictor of CHD mortality in SA patients (*P* < 0.001). Addition of GDF-15 improved the prognostic accuracy of a clinical risk prediction model concerning CHD mortality.

Neopterin	Estévez-Loureiro et al., 2009 [18]	Neopterin was found to be independent predictor of LV dysfunction in SA patients (*P* = 0.040).

IL-6	Tanindi et al., 2011 [22]	IL-6 levels were correlated with severe LAD stenosis (*P* < 0.001) and higher angiographic Gensini score (*P* < 0.001) in SA patients.

IL-10	Cavusoglu et al., 2011 [131]	Baseline elevated IL-10 levels were an independent predictor of adverse outcome in ACS patients.

IL-17	Liang et al., 2009 [43]	Significant correlation was found between plasma MPO and IL-17 levels in all study participants (*R* ^2^ = 0.9110, *P* < 0.05).

MPO	Liang et al., 2009 [43]	No significant difference between the control (24.2 ± 5.7 *μ*g/L) and SA groups (26.3 ± 4.8 *μ*g/L). MPO levels in patients with ACS (93.6 ± 20.3 *µ*g/L) were significantly higher than in patients with SA and the healthy control subjects (*P* < 0.05).

SDF-1; CXCL-12	Stellos et al., 2011 [58]	No correlation of SDF-1 with any biochemical parameter (except an inverse correlation with cholesterol levels, *P* = 0.035), either in the whole study population or in the SA group. No statistical difference in SDF-1 levels between NSTEMI and SA groups.

PCT	Sinning et al., 2011 [67]	Increased PCT levels in ACS group than in SA group (*P* for trend was *P* < 0.0001). Increased PCT levels at baseline were related to higher cardiovascular mortality (*P* = 0.00018) and higher cardiovascular event rate (*P* = 0.026). Also, independently related to future cardiovascular death (HR: 1.34; 95% CI: 1.08–1.65; *P* = 0.0070) when adjusted for clinical variables.

Fetuin-A	Bilgir et al., 2010 [70]	Decreased fetuin-A levels in SA group than in controls. Higher fetuin-A levels in SA patients, compared to AMI patients (1.67 ± 0.20 ng/mL versus 1.56 ± 0.21 ng/mL, *P* = 0.020).

Lp-PLA_2_	Ikonomidis et al., 2011 [79]	Major risk factor for CHD and also fatal cardiovascular events, mainly in lipidemic middle-aged men.

MMP-8, MMP-9	Jönsson et al., 2011 [89]	Both MMP-8 and MMP-9 levels did not correlate with clinical characteristics. No difference in serum or plasma levels of MMP-8/MMP-9 between SA patients and controls.

TIMP-1, TIMP-2	Brunner et al., 2010 [91], Fiotti et al., 2008 [94], Jönsson et al., 2011 [89]	No significant difference in TIMP-1/TIMP-2 levels between SA groups and controls.

Copeptin	Von Haehling et al., 2012 [107]	Higher baseline copeptin levels in patients with family CAD history. Patients with serum level ≥20.4 pmol/L suffered more events of the combined primary endpoint and of all-cause death in 90 days.

ACS: acute coronary syndrome; CHD: chronic heart disease; CAD: coronary artery disease; SA: stable angina; AMI: acute myocardial infarction; STEMI: ST-elevation myocardial infarction; NSTEMI: non-ST elevation myocardial infarction; LV: left ventricle; LAD: left anterior descending artery; CRP: C-reactive protein; GDF-1: growth differentiation factor-1; IL-6: interleukin-6; IL-10: interleukin-10; IL-17: interleukin-17; MPO: myeloperoxidase; SDF-1: stromal-cell derived factor-1; CXCL-12: C-X-C motif ligand 12; PCT: procalcitonin; MMP: matrix metalloproteinase; TIMP: tissue inhibitor of metalloproteinases.
